# Can Leaf Water Content Be Estimated Using Multispectral Terrestrial Laser Scanning? A Case Study With Norway Spruce Seedlings

**DOI:** 10.3389/fpls.2018.00299

**Published:** 2018-03-08

**Authors:** Samuli Junttila, Junko Sugano, Mikko Vastaranta, Riikka Linnakoski, Harri Kaartinen, Antero Kukko, Markus Holopainen, Hannu Hyyppä, Juha Hyyppä

**Affiliations:** ^1^Department of Forest Sciences, University of Helsinki, Helsinki, Finland; ^2^Centre of Excellence in Laser Scanning Research, Finnish Geospatial Research Institute (FGI), Masala, Finland; ^3^School of Forest Sciences, University of Eastern Finland, Joensuu, Finland; ^4^Natural Resources Institute Finland (Luke), Helsinki, Finland; ^5^Department of Remote Sensing and Photogrammetry, Finnish Geospatial Research Institute (FGI), Masala, Finland; ^6^Department of Geography and Geology, University of Turku, Turku, Finland; ^7^Department of Built Environment, Aalto University, Aalto, Finland

**Keywords:** terrestrial laser scanning, tree health, drought stress, multispectral laser scanning, leaf water content, forest damage, *Endoconidiophora polonica*

## Abstract

Changing climate is increasing the amount and intensity of forest stress agents, such as drought, pest insects, and pathogens. Leaf water content, measured here in terms of equivalent water thickness (EWT), is an early indicator of tree stress that provides timely information about the health status of forests. Multispectral terrestrial laser scanning (MS-TLS) measures target geometry and reflectance simultaneously, providing spatially explicit reflectance information at several wavelengths. EWT and leaf internal structure affect leaf reflectance in the shortwave infrared region that can be used to predict EWT with MS-TLS. A second wavelength that is sensitive to leaf internal structure but not affected by EWT can be used to normalize leaf internal effects on the shortwave infrared region and improve the prediction of EWT. Here we investigated the relationship between EWT and laser intensity features using multisensor MS-TLS at 690, 905, and 1,550 nm wavelengths with both drought-treated and *Endoconidiophora polonica* inoculated Norway spruce seedlings to better understand how MS-TLS measurements can explain variation in EWT. In our study, a normalized ratio of two wavelengths at 905 and 1,550 nm and length of seedling explained 91% of the variation (*R*^2^) in EWT as the respective prediction accuracy for EWT was 0.003 g/cm^2^ in greenhouse conditions. The relation between EWT and the normalized ratio of 905 and 1,550 nm wavelengths did not seem sensitive to a decreased point density of the MS-TLS data. Based on our results, different EWTs in Norway spruce seedlings show different spectral responses when measured using MS-TLS. These results can be further used when developing EWT monitoring for improving forest health assessments.

## Introduction

Measuring tree health is an increasingly important topic as the world's climate is warming and the human population is growing, putting the environment and forests under increasing stress (Williams et al., 2013). Stress and different disturbance events caused by, for example, insects are natural processes in forest ecosystems that can help maintain healthy and heterogeneous forests (Raffa et al., 2009). However, forests are facing new stresses and disturbance events such as drought, invasive pests, and air pollution, which are more intense than those they have previously adapted to (Trumbore et al., 2015). Forests provide many ecosystem services, such as carbon storage, recreational activities, timber, and non-timber forest products, which are jeopardized by declining forest health, increased forest mortality, and increased susceptibility to forest fires (Hanewinkel et al., 2013; McDowell and Allen, 2015; Morris et al., 2016). Managing declining forests efficiently in the face of climate change requires more information on the condition of forests to be able to monitor and control forest damage and fires (Allen et al., 2010); thus, new methods for mapping and monitoring forest health are needed.

Existing forest health mapping methods can be divided according to spatial, spectral, and temporal resolutions (Senf et al., 2017). Large-scale mapping of insect infestation has been conducted using single and multi-date Landsat data at 30 m resolution (Goodwin et al., 2008; Meddens et al., 2013). These methods are capable of capturing forest health information on a large scale and are suitable for countries where extensive forest management is applied, but spatially, more detailed approaches are necessary for capturing small-scale dynamics of forest decline in smaller countries with a more intensive approach to forest management. Spatially more accurate hyperspectral remote sensing data with 5 m resolution have been used to investigate the mapping of bark beetle induced tree mortality resulting in high overall accuracy (84–96%) in classifying dead trees (Fassnacht et al., 2014). Multispectral satellite imagery with 4 m resolution has been used to detect the red stage in mountain pine beetle infestation, resulting in high overall accuracy of 71–92% (White et al., 2005). However, none of the methods studied so far have been able to accurately detect early stages of bark beetle infestation and early tree stress when the trees have exhibited few visual symptoms (Fassnacht et al., 2014).

Leaf water content, typically measured as the weight of water per leaf area or equivalent water thickness (EWT), is an indicator of tree health that is affected by several tree stressors (Carter, 1993; Chaerle and Van Der Straeten, 2000). Drought, pest insects, and pathogens affect EWT, often before other symptoms appear, such as discoloration or defoliation; thus, detecting changes in EWT provides an early-warning signal of tree stress (Skakun et al., 2003; Zarco-Tejada et al., 2003). Leaf and canopy water content derived from Landsat data have been used as a proxy to detect large-scale insect infestation (Skakun et al., 2003; Wulder et al., 2006). Early detection of tree stress is crucial in providing timely information about the condition of forests, mitigating damage, and preventing the spread of the stressor (Wermelinger, 2004). For example, the European spruce bark beetle (*Ips typographus* L.) is a forest insect pest whose main host is Norway spruce trees. The bark beetle and a fungal pathogen have a mutual relationship causing disruptions in the flow of water and nutrients in the phloem and sapwood of the trees, resulting in decreased water content in the canopy and the sapwood (Horntvedt et al., 1983). Recently infested trees do not exhibit visual symptoms (green attack), but a reddish-brown coloring of tree crowns appears when the beetles have already left the host tree (Lausch et al., 2013). Thus, early detection of tree stress would greatly assist in mitigating and managing the damage caused by *I. typographus*. Efforts have been made to solve this issue but none have succeeded with reasonable accuracy thus far (Lausch et al., 2013; Fassnacht et al., 2014).

Improving reference data for assessing forest health has been identified as a key research area in need of development (Senf et al., 2017). Because field reference data or the ground truth is the gold standard of modeling, any errors in the data can lead to significant bias and errors in modeling. The high costs of collecting field data usually lead to a compromise between the number of plots and the spatial extent of the investigation. National forest inventories may collect forest health data, but forest inventory data are not available in all countries (Gschwantner et al., 2016). Visual assessment of forest health in field data collection is prone to error and bias due to the subjective nature of the estimation, and it requires substantial expertise. The early stages of tree stress can be visually difficult to determine because trees may not exhibit visual symptoms at this stage. Thus, objective and reliable methods for collecting forest health related reference data in the field are needed.

Equivalent water thickness is closely related to another metric for calculating vegetation water content—fuel moisture content (FMC)—that is more frequently used in forest fire danger literature (Chuvieco et al., 2004). Fuel moisture content is expressed as the ratio between the weight of water and sample dry weight and is an important parameter in fire ignition (Dimitrakopoulos and Papaioannou, 2001). A multitemporal analysis of NOAA-AVHRR images has been used to derive FMC for Mediterranean grasslands and shrub species with *R*^2^-values over 0.8 (Chuvieco et al., 2004). Generally, studies aiming to estimate FMC use spatially coarse resolution satellite data that can provide frequent imagery, important in forest fire danger estimation (Sow et al., 2013).

Multispectral terrestrial laser scanning (MS-TLS) measures the range and reflectance of the target at several wavelengths simultaneously, providing three-dimensional estimates of reflectance. Several MS-TLS instruments have been developed recently mainly for laboratory use (Douglas et al., 2012; Hakala et al., 2012; Wallace et al., 2012; Wei et al., 2012; Niu et al., 2015; Li et al., 2016). These instruments utilize a supercontinuum laser source that is powerful enough to not be eye-safe, limiting their operation in the field, with the exception of a terrestrial laser scanner operating at two wavelengths, which is also portable (Douglas et al., 2012). Recently, a commercial multispectral laser scanner—the Optech Titan (Teledyne Optech, Canada)—with three channels has become available for airborne measurements (Matikainen et al., 2016). The development of multispectral laser scanning instruments widens the possibilities of using laser scanning data as more information about the target's reflectance becomes available in a three-dimensional format.

Alterations in EWT affect leaf reflectance in the shortwave infrared region (Ceccato et al., 2001), which can be measured using MS-TLS. A number of studies have investigated the use of MS-TLS and terrestrial laser scanning (TLS) in the estimation of EWT. Gaulton et al. (2013) studied the potential of estimating leaf EWT with a dual-wavelength terrestrial laser scanner at 1,063 and 1,545 nm wavelengths, resulting in a significant correlation (*R*^2^ = 0.8, root mean square error [RMSE] = 0.0069 g/cm^2^) between leaf EWT and a normalized ratio of the two wavelengths, but with a low number of samples and species. The estimation of leaf EWT has been recently studied using MS-TLS at 690 and 1,550 nm wavelengths with single leaves, resulting in a strong correlation (*R*^2^ = 0.93, RMSE = 0.004 g/cm^2^) between EWT and a normalized ratio of the two wavelengths, but the selection of wavelengths was concluded to be suboptimal (Junttila et al., 2016). Zhu et al. (2015) showed that TLS intensity data at 1,550 nm were able to explain 76% of the variation in EWT at leaf level after radiometric correction of the TLS intensity data. Canopy EWT has been estimated with full-waveform single wavelength TLS at 1,550 nm wavelength, resulting in a significant correlation between canopy EWT and backscatter coefficient (*R*^2^ = 0.66, RMSE = 0.001 g/cm^2^) (Zhu et al., 2017). However, single wavelength data seem to be sensitive to confounding factors, such as incidence angle, requiring complicated correction of the intensity data, and they are also affected by structural variables of the leaf other than EWT (Zhu et al., 2015, 2017). The requirement of an incidence angle correction limits the utilization of the single wavelength method to deciduous species with distinguishable leaves. Coniferous forests comprise a large part of the world's forest biomes, but thus far only Junttila et al. (2016) have looked into the estimation of EWT with coniferous species using MS-TLS. The use of spectral ratios calculated from dual-wavelength intensity data has been shown to be insensitive to incidence angle effects thus enabling higher accuracy in the estimation of EWT (Hancock et al., 2017). A second wavelength in the near infrared region coupled with a wavelength in the shortwave infrared region is also capable of normalizing leaf structural effects on the estimation of EWT (Ceccato et al., 2001).

The aim of this study was to investigate the capability of multisensor MS-TLS in monitoring leaf EWT, with Norway spruce (*Picea abies* L.) seedlings. First, the dependencies between EWT and laser intensity features at different wavelengths (and calculated spectral ratios) of segmented point clouds are examined to find explanatory variables for predicting EWT. Secondly, we compare the combination of 690, 905, and 1,550 nm wavelengths in predicting EWT using different sensors. Thirdly, the importance of point density in estimating EWT is evaluated using random sampling of the point clouds to decrease the point density. This research contributes to the first steps in developing a method for the accurate estimation of leaf EWT in forests using MS-TLS.

## Materials and methods

### Experiment design

Two-year-old commercial Norway spruce seedlings (*n* = 145) were grown for 12 weeks in a greenhouse between May and August 2016. The seedlings were subjected to different treatments to induce drought and variation in EWT. The seedlings were divided into five groups for different treatments. Three of the groups received 75% (28 ml), 50% (20 ml), and 25% (12 ml) of “normal” watering (watered as required to maintain moist soil) three times a week (groups D75, D50, and D25, respectively) (Table 1). The fourth group was grown with a sufficient amount of water (36 ml) for 10 weeks until irrigation was completely stopped, and the seedlings then went without water for 3 weeks (group D_total). The fifth group of seedlings was inoculated with *Endoconidiophora polonica* (isolate CBS 142283), a fungal pathogen associated with the European spruce bark beetle (*I. typographus*) that disturbs the flow of water and nutrients in the phloem and sapwood (group F). The amount of water given to the seedlings in the drought treatment groups (D75, D50, and D25) was adjusted during the experiment because the seedlings showed resistance to drought (Table 2). After 34 days from the start of the experiment, irrigation was reduced to twice a week; after 46 days, the amount of water given was cut to half; and finally after 56 days from the start of the experiment the irrigation was reduced to once a week. During the 12 weeks, 5–16 seedlings were randomly collected for TLS measurements at eight time intervals. Two or three seedlings were collected from each of the drought treatment groups (D75, D50, and D25) and eight seedlings from the fungal treatment group (F) for each MS-TLS measurement. Seedlings that showed severe symptoms during the final stages of the experiment, and which were unlikely to survive until the next measurement time were prioritized to avoid the loss of research material, resulting in uneven sample numbers toward the end of the experiment. Details of the measurements can be found in Table 3. The mean length of the seedlings was 30.3 cm (std ± 5.34 cm) during the experiment.

**Table 1:** Treatment groups and related statistics.

**Group name**	**Irrigation amount per seedling (ml)**	**Number of seedlings**
D75	28	25
D50	20	23
D25	12	23
D_total	36 (until stopping)	23
F	36	51

**Table 2:** Irrigation of the drought experiment groups.

**Dates**	**Irrigation amount per seedling (ml) for each group**			**Irrigation times per week**
	**D75**	**D50**	**D25**	
June 19–June 23	28	20	12	3
June 24–July 5	28	20	12	2
July 6–July 15	14	10	6	2
July 16–August 18	14	10	6	1

**Table 3:** EWT for each measurement and treatment group, and their respective statistics.

**Measurement date**	**Days from starting the experiment**	**Group name**	**Measurements**	**Mean EWT (g/cm^2^)**	**Min EWT (g/cm^2^)**	**Max EWT (g/cm^2^)**	**Standard deviation of EWT (g/cm^2^)**
**EWT**							
May 19	0	D75	3	0.035	0.033	0.038	0.0032
May 19	0	D50	2	0.035	0.031	0.039	0.0061
May 19	0	D25	3	0.031	0.027	0.038	0.0055
May 19	0	F	8	0.033	0.027	0.040	0.0040
June 3	15	D75	2	0.037	0.034	0.039	0.0035
June 3	15	D50	3	0.026	0.031	0.038	0.0060
June 3	15	D25	3	0.029	0.028	0.030	0.0007
June 3	15	F	8	0.016	0.006	0.032	0.0092
June 9	21	D75	2	0.032	0.032	0.032	0.0004
June 9	21	D50	3	0.036	0.029	0.043	0.0072
June 9	21	D25	2	0.032	0.030	0.033	0.0020
June 9	21	F	8	0.016	0.004	0.031	0.0110
June 17	29	D75	2	0.032	0.029	0.035	0.0037
June 17	29	D50	2	0.029	0.029	0.030	0.0004
June 17	29	D25	3	0.030	0.026	0.035	0.0046
June 17	29	F	9	0.025	0.007	0.032	0.0091
June 23	35	D75	2	0.034	0.030	0.038	0.0055
June 23	35	D50	3	0.032	0.029	0.034	0.0025
June 23	35	D25	3	0.032	0.028	0.037	0.0043
June 23	35	F	8	0.028	0.013	0.033	0.0073
July 5	47	D50	2	0.029	0.029	0.030	0.0009
July 5	47	D25	4	0.027	0.024	0.030	0.0025
July15	57	D75	4	0.029	0.026	0.032	0.0030
July 15	57	D50	3	0.026	0.020	0.031	0.0056
July 15	57	D25	4	0.018	0.015	0.025	0.0049
July 15	57	F	3	0.038	0.036	0.039	0.0016
July 22	64	D75	9	0.017	0.010	0.025	0.0058
July 22	64	D50	4	0.023	0.014	0.033	0.010
July 22	64	D25	1	0.022	0.022	0.022	–
July 22	64	F	2	0.040	0.039	0.040	0.0004
August 18	81	D75	1	0.003	0.003	0.003	–
August 18	81	D50	1	0.004	0.004	0.004	–
August 18	81	D_total	28	0.012	0.003	0.032	0.0058

### Terrestrial laser scanners

The seedlings were scanned consecutively with three different terrestrial laser scanners from the same position from a distance of 5.2 m inside the greenhouse. The terrestrial laser scanners used were a Leica HDS6100 (Leica Geosystems AG, Heerbrugg, Switzerland), a FARO S120 (FARO Europe GmbH & Co. KG, Korntal-Münchingen, Germany), and a FARO X330, utilizing wavelengths of 690, 905, and 1,550 nm, respectively. The technical specifications of the terrestrial laser scanners can be found in Table 4. The varying beam diameter at exit and beam divergence resulted in different spot sizes at target distance, which were 4.14, 3.99, and 3.24 mm for the Leica, the S120, and the X330, respectively. The scanning was done using the highest resolution available for each instrument, resulting in a point spacing of ~1 mm; thus, there was significant overlap between consecutive laser measurements.

**Table 4:** Technical specifications for the terrestrial laser scanners.

**Scanner type**	**Beam divergence (mrad)**	**Beam diameter at exit (mm)**	**Wavelength (nm)**	**Output power (mW)**	**Scan rate (kHz)**	**Intensity recording (DN)**	**Ranging error (mm)**
Leica HDS6100	0.22	3	690	30	508	−1,228 to 2,048	±2
FARO S120	0.19	3	905	20	488	−2,048 to 2,033	±2
FARO X330	0.19	2.25	1,550	500	488	−2,048 to 2,033	±2

*DN, digital number*.

Three white spheres were used as common targets to register the scans to the same coordinate system as each other, facilitating the segmentation of point clouds in the processing of the data. A four-grade Spectralon (Labsphere, North Sutton, NH, USA) panel was used as a reference target for intensity normalization. The size of the panel was 460 mm x 460 mm consisting of four different reflectance panels (nominal reflectances of 99, 50, 25, and 12%), each sized 115 × 460 mm. Approximately 65,000 points were received from each of the panels.

### Ecophysiological measurements

After scanning, the seedlings were transported to the laboratory for EWT measurements (Table 3). A sample of 30–40 needles was randomly collected from each seedling, constituting a single measurement of EWT. As the seedlings were growing during the experiment, only previous year needles were sampled to avoid the influence of needle development on the EWT measurements. The needles were weighed (with a precision of 0.0001 g) to measure fresh weight, scanned with an Epson V370 Photo Scanner (Epson America, Inc., CA, USA) at 800 dpi resolution to measure leaf area, and dried in an oven at 60°C for 48 h to measure dry weight. The images were analyzed with the open source software EasyLeafArea (Easlon and Bloom, 2014) to segment the needles and calculate leaf area based on the number of segmented pixels. The EWT was then calculated according to Danson et al. (1992):

(1) $$\begin{array}{r} \frac{FW-DW}{A}\left(\frac{g}{cm^{2}}\right), \end{array}$$

where FW is the fresh weight of the needles (g), DW is the weight of the dried needles (g), and A is the surface area of the fresh needles (cm^2^).

### Terrestrial laser scanning data processing

The TLS scans were registered to a common coordinate system using the Z + F LaserControl software package (Zoller + Fröhlich GmbH, Wangen im Allgäu, Germany), resulting in a mean accuracy of 2.7 mm between targets. The registration process was based on Helmert transformation and three sphere-shaped targets with known locations (Watson, 2006). The registration of the scans was visually inspected after the registration process to ensure the alignment of the point clouds at the range of the seedlings. Because the terrestrial laser scanners utilize a phase shift measurement technique to determine the range to the target, a large number of ghost points were produced in the scans (Balduzzi et al., 2011). To reduce the number of ghost points, the point clouds were filtered with a statistical outlier algorithm based on the distance between points by using the CloudCompare v.2.8.1 (Girardeau-Montaut, 2011) software. The algorithm calculates the maximum distance for a point to be included using the following equation:

(2) $$\begin{array}{r} MaxD=MeanD+nSigma*std, \end{array}$$

where *MaxD* is the maximum distance for a point to be included, *MeanD* is the mean distance of the neighboring points, *nSigma* is a standard deviation multiplier threshold, and *std* is the standard deviation of the distance of the neighboring points. Thus, the algorithm requires two parameters: the number of neighbors and *nSigma*. The parameters were determined though iteration and visual estimation of the result. Fifty neighbors were used for every scan and the *nSigma* was set to 1.0 and 0.65 for the Leica and FARO scanners respectively.

Then, cloud-to-cloud distances were calculated for the point clouds in the CloudCompare software package where the FARO X330 was used as a reference cloud for calculating the distances because of its expected sensitivity to EWT (Junttila et al., 2016). The tool calculates the distance of each point to the nearest point in the reference cloud. A maximum distance of 2 mm (which is also the ranging accuracy of the terrestrial laser scanners) was used to filter the point clouds and match the geometry of the point clouds produced by different terrestrial laser scanners with each other.

Points from each seedling were then manually clipped from the georeferenced point clouds for further processing. The point clouds from each seedling were further manually segmented into stem and leaf points to evaluate their effect on the estimation of EWT.

Because the point clouds were produced using a very high-resolution scanning setting, the point clouds were randomly sampled to reduce the amount of points in each point cloud and investigate how the point density affects the estimation of EWT. The point clouds were randomly sampled to four different point cloud sizes: 2,000, 1,000, 500, and 250 points per seedling. The mean number of points for each seedling was 14,702 in the unsegmented point clouds; thus, the amount of points was greatly reduced.

### Intensity calibration

Terrestrial laser scanners are primarily designed for range measuring; thus, they are not optimized for reflectivity measurements. Some terrestrial laser scanners have been shown to have a nonlinear intensity scale (Kaasalainen et al., 2011); thus, the calibration of intensity data may be required. The stationary four-grade Spectralon panel was used as a reference target for the calibration procedure using the mean of ~65,000 points from each of the reflectance panels. The standard deviation of raw intensity values of these samples varied between 4.7 and 8.9. The panel's reflectance was also measured with a FieldSpec Pro FR (Analytical Spectral Devices, Inc., Longmont, CO, USA) field spectrophotometer. The relationship between the spectrophotometer measured reflectance and the laser scanner measured intensity was then investigated to detect any nonlinearities in the intensity response.

**Figure 1:**
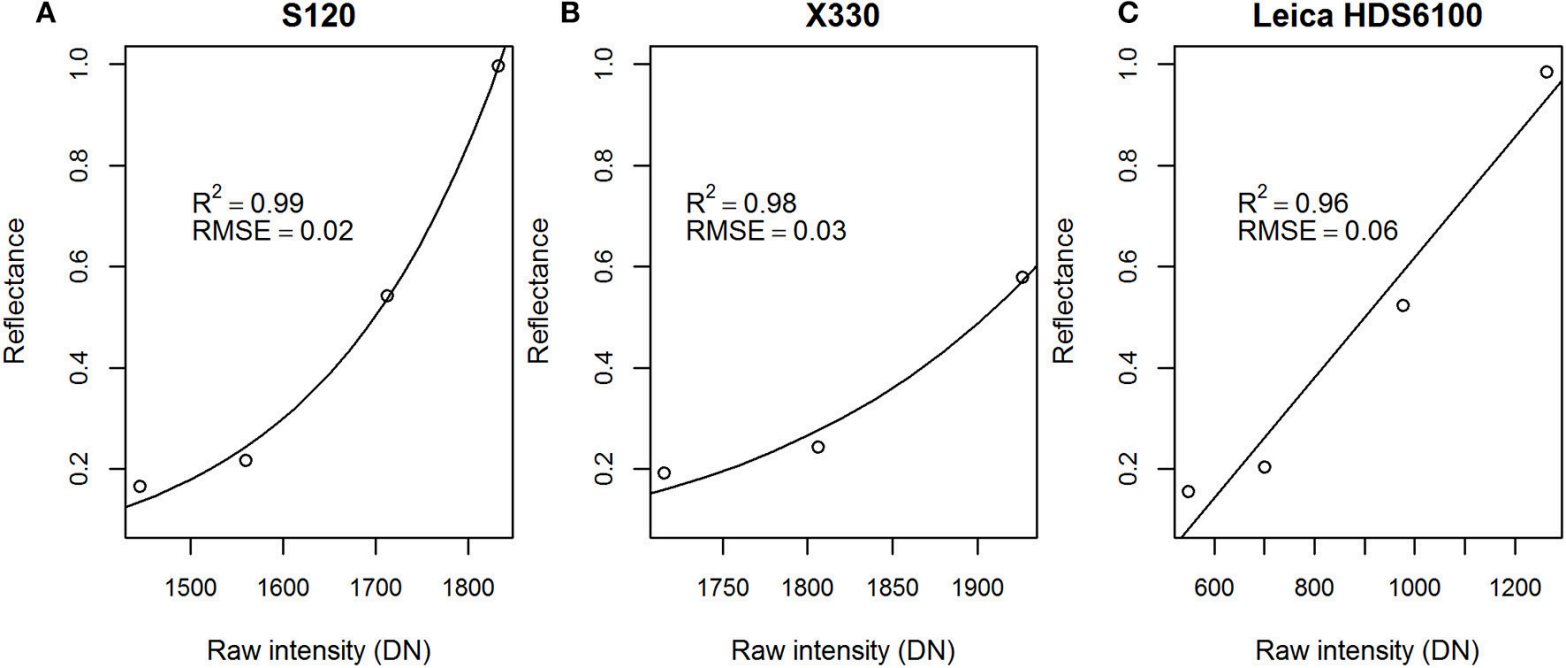
Regression models for reflectance and measured raw intensity for the **(A)** FARO S120, **(B)** FARO X330, **(C)** and Leica scanners, and their coefficients of determination (R^2^) and root mean square error (RMSE) values. DN, digital number.

The relationship between measured intensity and reflectance was shown to be linear for the Leica scanner and logarithmic for the FARO scanners (Figure 1). The measured intensity from the FARO X330 scanner was shown to be saturated in the 99% panel because the maximum intensity of the digital number was reached; thus, the calibration of FARO X330 data was done using only the 50, 25, and 12% panels. To correct for the logarithmic effect of the FARO scanners, Equation 3 was used (Kaasalainen et al., 2009):

(3) $$\begin{array}{r} y=10^{\frac{\left(x-A_{1}\right)}{A_{0}}}, \end{array}$$

where *A*_0_ and *A*_1_ are empirical parameters determined by fitting the raw intensity measured by the FARO scanners to the spectrophotometer measured reflectance. Additionally, a linear regression model was developed to model the relationship between the raw intensity and reflectance for the Leica scanner to convert the raw intensity values to calibrated intensity. The fitted parameter values of *A*_1_ and *A*_0_ were 1833.8 and 446.9 for FARO S120, and 2018.7 and 379.9 for FARO X330. The linear regression model of the Leica scanner had a coefficient of 0.00119 and an intercept of −0.57186.

The effect of ambient temperature, temperature of the scanner, and background illumination on measured intensity was then reduced by normalizing each scan with the mean intensity of ~20,000 returns from the 99% Spectralon panel in the case of the Leica and FARO S120, and from the 50% Spectralon panel for the FARO X330 due to the saturation of intensity values in the 99% panel. The intensity of the X330 points from the seedlings had values below the mean of the 50% Spectralon panel; thus, the intensity was within the calibrated range.

The result of this process is referred to as the calibrated intensity within this paper. Because the samples were scanned within a narrow range bin of less than 1 m, no range corrections were conducted. A range correction is required when the targets are located at different distances from the scanner, i.e., scanning trees in a forest environment (Kaasalainen et al., 2011).

### Laser intensity features

A set of statistical features was calculated from the intensity data from each point cloud representing a seedling at each wavelength. Intensity features were calculated from the calibrated intensity data of each point cloud. These features were the mean, minimum, maximum, standard deviation and percentiles 10, 20, 30, 40, 60, 70, 80, and 90 (*p*-value, e.g., p20, p60) of the intensity value distribution. Within this paper these statistics are referred to with a wavelength and a subscript describing the feature (e.g., 1,550_mean_, 690_p30_). The included features are shown in Table 5.

**Table 5:** Intensity features.

**Feature**	**Abbreviation**	**Description**
Mean	mean	Mean calibrated intensity
Standard deviation	std	Standard deviation of calibrated intensity
Percentile	p…i	ith percentile of the intensity value distribution
Minimum	min	Minimum calibrated intensity
Maximum	max	Maximum calibrated intensity

Based on these features, a set of spectral indices was calculated for each point cloud (Table 6). We aimed to observe combinations of the 690 and 1,550 nm and 905 and 1,550 nm wavelengths based on ratio and normalization operations by calculating a simple ratio (SR) index (Jordan, 1969) and a normalized difference index (NDI) (Hancock et al., 2017) as follows:

(4) $$\begin{array}{r} \text{SR}\_i_{\rho 1,\rho 2}=\frac{\rho 1_{i}}{\rho 2_{i}}, \end{array}$$

(5) $$\begin{array}{r} \text{NDI}\_i_{\rho 1,\rho 2}=\frac{\rho 1_{i}-\rho 2_{i}}{\rho 1_{i}+\rho 2_{i}}, \end{array}$$

where ρ1_i_ and ρ2_i_ are the *i* feature of wavelength ρ1 and ρ2.

**Table 6:** Calibrated intensity values for the mean of each wavelength and each spectral index for all points, leaf points, and stem points.

**Laser intensity feature**	**All points**				**Leaf points**				**Stem points**			
	**Mean**	**Min**	**Max**	**Std**	**Mean**	**Min**	**Max**	**Std**	**Mean**	**Min**	**Max**	**Std**
690_mean_	0.16	0.10	0.27	0.03	0.14	0.08	0.27	0.04	0.33	0.18	0.43	0.04
905_mean_	0.16	0.11	0.25	0.03	0.14	0.10	0.25	0.03	0.35	0.29	0.42	0.03
1,550_mean_	0.09	0.07	0.12	0.02	0.08	0.06	0.11	0.01	0.20	0.14	0.32	0.04
SR_mean_690, 1550_	1.70	0.84	2.69	0.43	1.64	0.82	2.53	0.44	1.69	0.76	2.35	0.31
SR_mean_905, 1550_	1.75	1.26	2.31	0.30	1.75	1.19	2.35	0.33	1.73	1.25	2.44	0.24
NDI_mean_690, 1550_	0.24	−0.09	0.46	0.12	0.22	−0.10	0.43	0.13	0.24	−0.14	0.40	0.10
NDI_mean_905, 1550_	0.26	0.11	0.39	0.08	0.26	0.09	0.40	0.09	0.26	0.11	0.42	0.07

*NDI, normalized difference index; SR, simple ratio (index)*.

The indices are referred to with the abbreviation, feature name, and a subscript describing the wavelengths used for the calculation (e.g., NDI_p80_690, 1,550_). The total number of calculated laser intensity features was 48.

### Statistical analysis

The Student's *t*-test was used to determine whether there were statistically significant differences in the length of the seedlings in the different treatment groups. The relationship between EWT and each laser intensity feature was investigated using simple linear regression. The regression models were developed for all points, leaf points and stem points separately. Regression models were also developed for the randomly sampled point clouds to investigate how point density affects the estimation of EWT. Multiple regression was used to investigate whether the estimation of EWT could be improved by including length of the seedling as a structural variable in the regression model with the best explanatory laser intensity feature. We used the coefficient of determination (*R*^2^) and RMSE with cross-validation to assess the goodness of fit between the dependent and the predictor using the following equations:

(6) $$\begin{array}{r} RMSE=\sqrt{\frac{\overset{n}{\underset{\text{i}=1}{\sum }}{\left(y_{\text{i}}-{ŷ}_{\text{i}}\right)}^{2}}{n}}, \end{array}$$

(7) $$\begin{array}{r} R^{2}=1-\frac{\sum_{\text{i}}{\left(y_{\text{i}}-{ŷ}_{\text{i}}\right)}^{2}}{\sum_{\text{i}}{\left(y_{\text{i}}-ȳ\right)}^{2}}, \end{array}$$

where *n* is the number of observations, *y*_*i*_ is the observed value for the measurement *i*, ŷ_*i*_ is the predicted value for the measurement *i*, and ȳ is the mean of the observed data. All of the statistical analyses were performed using the open source software package R ver. 3.2.3 (R Core Team, 2013). Due to the large number of explanatory laser intensity features, only the 10 best explaining variables with the highest *R*^2^ were reported.

## Results

### Effects of drought on growth and equivalent water thickness

**Figure 2:**
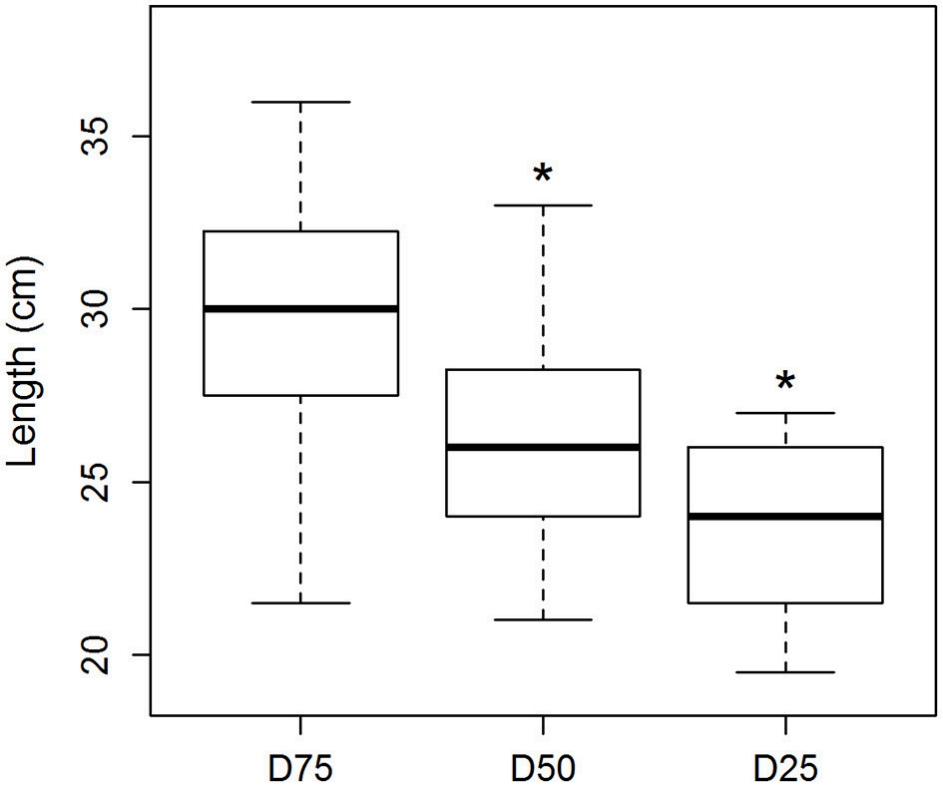
Length of the seedlings in the drought treatment groups (number of samples: D75: 21, D50: 17, D25: 13). Asterisk (^*^) on top of the bar denotes statistically significant difference between the groups. Black line denotes the median value, upper and lower edge of the box show 75 and 25% percentiles, respectively. The whiskers of the boxes extend to the extreme values of the data sets.

The drought treatments resulted in varying lengths of the seedlings in the treatment groups (Figure 2). The D75 group had a mean length of 29.5 cm, while the D50 and D25 groups had significantly lower mean lengths of 26.0 and 23.7 cm, respectively. The drought treatments significantly affected the growth of the seedlings.

**Figure 3:**
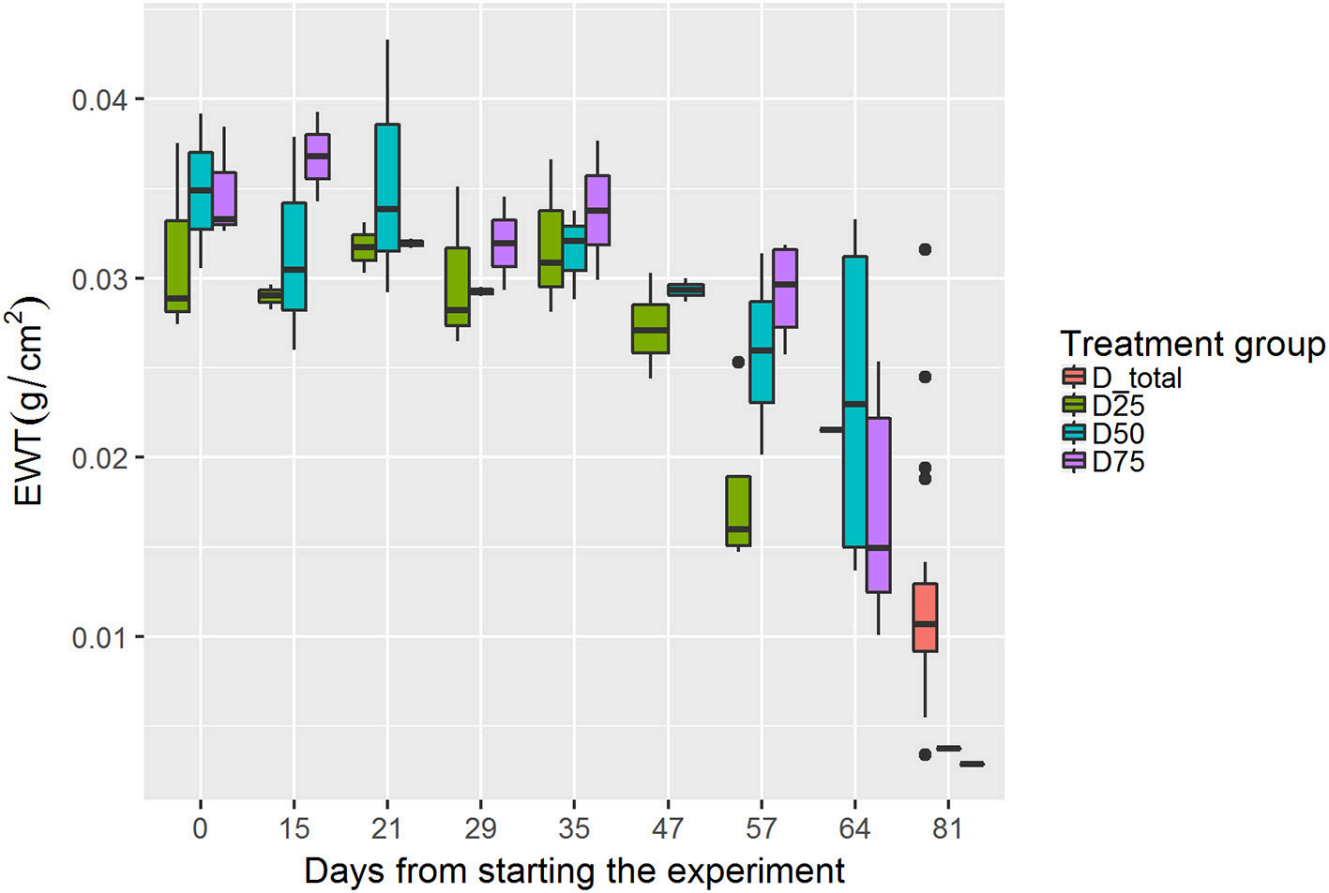
Changes in EWT in each drought treatment group during the experiment (each bar represents approximately 4 samples). Black line denotes the median value, upper and lower edges of the boxes show 75 and 25% percentiles, respectively. The whiskers of the boxes extend to extreme values no longer than 1.5 times the interquartile range. Values further than that are plotted as outliers.

The seedlings showed resistance to drought. Despite the different amounts of water that the seedlings in the drought treatment groups received, EWT was shown to stay at relatively similar levels between treatment groups until the irrigation amount was further reduced after 35 days from starting the experiment (Figure 3). The seedlings still showed few visual symptoms due to drought, and after further reductions of irrigation the EWT decreased significantly. The lowest EWT levels in the experiment were found at the end of the experiment in the D_total group.

### Effects of *E. polonica* treatment on equivalent water thickness

**Figure 4:**
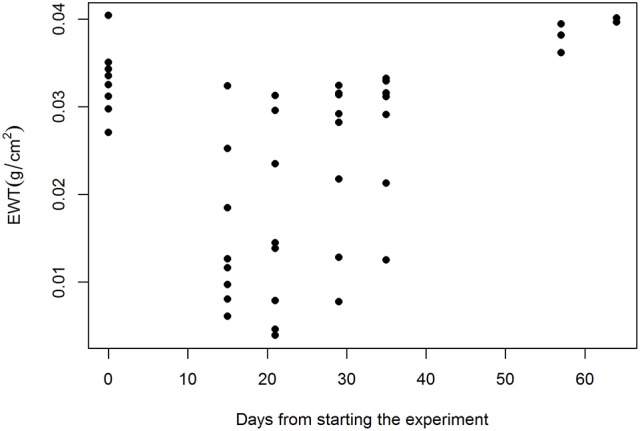
EWT alterations during the experiment in the fungal pathogen treatment group.

The fungal pathogen treatment of the seedlings resulted in decreased EWT (Figure 4). The affected seedlings showed a rapid decrease in EWT because the effects were apparent only 14 days after the inoculation. The infected seedlings were shown to respond to the treatment during the first 35 days of the experiment. After that period, no signs of infection in the remaining seedlings were observed.

### Does equivalent water thickness affect the spectral response of multispectral terrestrial laser scanning?

**Figure 5:**
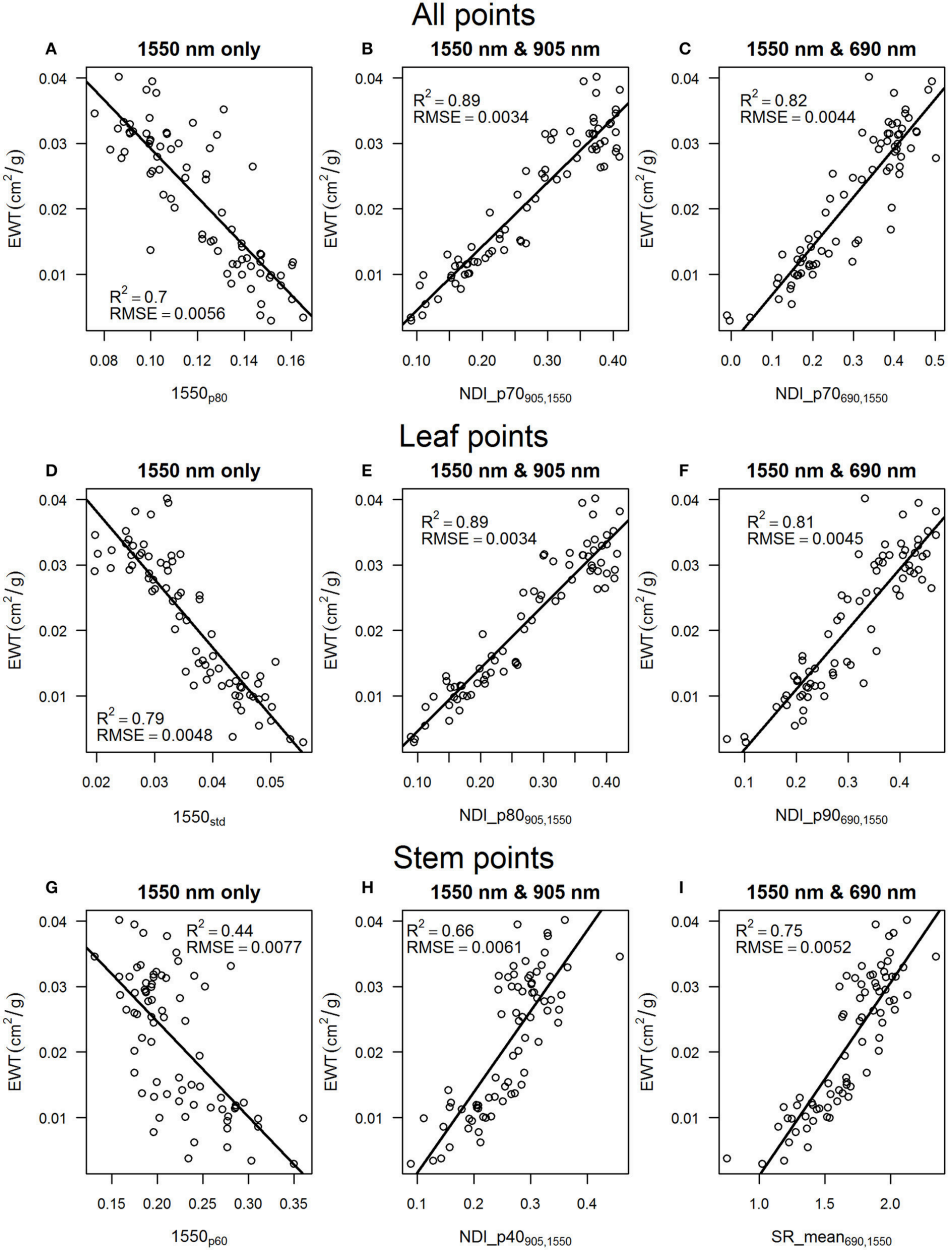
Relationships between EWT and the best explanatory laser intensity features with their respective *R*^2^ and RMSE values for all points for **(A)** 1,550 nm wavelength, **(B)** 1,550 and 905 wavelengths, **(C)** 1,550 and 690 nm wavelengths. The same for leaf points for **(D)** 1,550 nm wavelength, **(E)** 1,550 and 905 nm wavelengths, **(F)** 1,550 and 690 nm wavelengths, and the same for stem points for **(G)** 1,550 nm wavelength, **(H)** 1,550 and 905 nm wavelengths, **(I)** 1,550 and 690 nm wavelengths.

The calibrated intensity at 1550 nm wavelength from the FARO X330 showed significantly increasing values with decreasing EWT (Figure 5). The linear regression model of EWT and the best explanatory variable at 1,550 nm wavelength, 1,550_p80_, had a coefficient of determination of 0.7. For comparison, 1,550_mean_ was able to explain only 47% of the variation in EWT. Spectral indices calculated from both combinations −905 and 1,550 nm and 690 and 1,550 nm – were able to predict EWT with improved accuracy. The combination of 905 and 1,550 nm showed the best prediction accuracy of EWT with *R*^2^ of 0.89 and 0.87 for NDI_p70_905, 1,550_ and NDI_p60_905, 1,550_, respectively (Table 7). Similar prediction accuracy, with an *R*^2^ of 0.87, was also observed for SR_p70_905, 1,550_ and NDI_p80_905, 1,550_. The best explanatory features for the combination of 690 and 1,550 nm showed prediction accuracies of *R*^2^ of 0.82 and 0.80 for NDI_p70_690, 1,550_ and SR_p70_690, 1,550_, respectively.

**Table 7:** R^2^ and RMSE values for the linear regression models of equivalent water thickness and laser intensity features for all points, leaf points, and stem points.

**All points**			**Leaf points**			**Stem points**		
**Laser intensity feature**	**R^2^**	**RMSE (g/cm^2^)**	**Laser intensity feature**	**R^2^**	**RMSE (g/cm^2^)**	**Laser intensity feature**	**R^2^**	**RMSE (g/cm^2^)**
NDI_p70_905, 1,550_	0.89	0.0034	NDI_p80_905, 1,550_	0.89	0.0034	SR_mean_690, 1,550_	0.75	0.0052
NDI_p60_905, 1,550_	0.87	0.0036	NDI_p70_905, 1,550_	0.89	0.0035	SR_p40_690, 1,550_	0.74	0.0053
SR_p70_905, 1,550_	0.87	0.0037	SR_p70_905, 1,550_	0.87	0.0037	SR_p30_690, 1,550_	0.73	0.0054
NDI_p80_905, 1,550_	0.87	0.0037	SR_p80_905, 1,550_	0.87	0.0038	SR_p60_690, 1,550_	0.73	0.0054
SR_p60_905, 1,550_	0.86	0.0038	NDI_p60_905, 1,550_	0.86	0.0038	SR_p70_690, 1,550_	0.71	0.0056
NDI_mean_905, 1,550_	0.85	0.0040	NDI_p90_905, 1,550_	0.86	0.0039	NDI_mean_690, 1,550_	0.70	0.0056
SR_p80_905, 1,550_	0.84	0.0041	NDI_mean_905, 1,550_	0.85	0.0040	SR_p20_690, 1,550_	0.70	0.0057
SR_mean_905, 1,550_	0.83	0.0043	SR_p60_905, 1,550_	0.84	0.0041	NDI_p30_690, 1,550_	0.67	0.0059
NDI_p70_690, 1,550_	0.82	0.0044	SR_p90_905, 1,550_	0.83	0.0043	NDI_p60_690, 1,550_	0.67	0.0059
SR_p70_690, 1,550_	0.80	0.0046	SR_mean_905, 1,550_	0.82	0.0043	NDI_p40_690, 1,550_	0.67	0.0059

The best explanatory laser intensity features varied slightly for leaf points. The 1,550_std_ feature could explain 79%, and 1,550_p90_ 73%, of the variation in EWT as the best predicting features for single wavelength data. The spectral indices showed similar explanatory power compared with using all points, but the best features for estimating EWT were higher intensity percentiles than for all points. The NDI_p80_905, 1,550_ and the NDI_p70_905, 1550_ features explained 89% of the variation in EWT, while SR_p70_905, 1,550_ and SR_p80_905, 1,550_ explained 87% of the variation in EWT. The NDI_p90_690, 1,550_ and the SR_p90_690, 1,550_ features were the best predictors for the combination of 690 and 1,550 nm wavelengths, with *R*^2^ of 0.81 and 0.79, respectively.

The stem points were also shown to have statistically significant power in explaining the differences in EWT but with lower percentiles of the intensity value distribution and lower coefficients of determination of 0.49, 0.66, and 0.75 for 1550 nm only, 1,550 and 905 nm, and 1,550 and 690 nm, respectively. Single wavelength data explained 49% of the variation in EWT with the 1,550_mean_ feature. The combination of 690 and 1,550 nm wavelengths showed the highest prediction accuracy, with the SR_mean_690, 1,550_ and SR_p40_690, 1,550_ features resulting in an *R*^2^ of 0.75 and 0.74, respectively. The NDI_p40_905, 1,550_ and the NDI_p10_905, 1,550_ features were able to predict EWT with *R*^2^ of 0.66 and 0.65, respectively.

The regression models explained 89% of the variation in EWT when 2,000 points from each seedling were randomly sampled. Thus, there was only a 0.0001 g/cm^2^ increase in RMSE for both spectral indices compared using all points (Table 8). A slight decrease of 0.02 in *R*^2^ and an increase of 0.0002 g/cm^2^ was present for NDI_p70_905, 1,550_ after random sampling of 1,000 points, whereas NDI_p70_690, 1,550_ showed no changes compared with 2,000 points. Reducing the number of points to 500 resulted in no changes in the estimation accuracy of EWT for NDI_p70_905, 1,550_ but *R*^2^ decreased to 0.79 and RMSE increased to 0.0048 g/cm^2^ for NDI_p70_690, 1,550_. When the number of points was reduced to 250, *R*^2^ decreased to 0.79 and 0.78 and RMSE increased to 0.0047 g/cm^2^ and 0.0049 g/cm^2^ for NDI_p70_905, 1,550_ and NDI_p70_690, 1,550_, respectively.

**Table 8:** R^2^ and RMSE values for the linear regression models of the spectral indices and equivalent water thickness after random sampling of the point clouds.

**Spectral index**	**Number of points/seedling**	**R^2^**	**RMSE**
NDI_p70_905. 1,550_	2,000	0.89	0.0035
	1,000	0.87	0.0037
	500	0.87	0.0037
	250	0.79	0.0047
NDI_p70_690, 1,550_	2,000	0.81	0.0045
	1,000	0.81	0.0045
	500	0.79	0.0048
	250	0.78	0.0049

**Figure 6:**
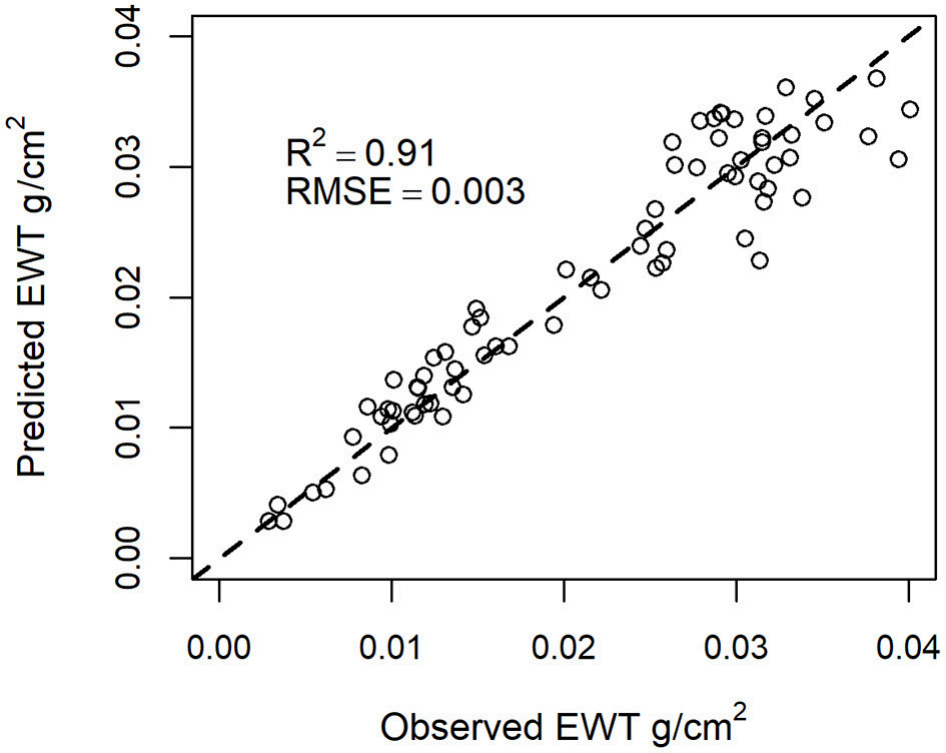
EWT estimation using NDI_p70_905, 1,550_ and length of the seedling as predictors.

Multiple regression was able to improve the prediction of EWT. According to the multiple regression analysis, the length was statistically significant at *p* < 0.001. The estimation accuracy of EWT was improved compared with using simple regression models, with *R*^2^ = 0.91 and RMSE = 0.003 g/cm^2^ (Figure 6).

## Discussion

The seedlings showed surprisingly high resistance to drought despite the low amounts of irrigation for each seedling. The drought led to a significant reduction in growth, which correlated with the intensity of the drought treatment. The seedlings seemed to cope with drought by reducing growth, which led to reduced demand for water because there was less foliage to support and less transpiring leaf area. However, significant reduction in EWT was observed after further reduction in irrigation.

The results showed that the investigated laser intensity features were able to explain most of the variation in leaf EWT and provide a relatively good prediction accuracy. Single wavelength intensity features at 1,550 nm were able to explain 70–79% of the differences in EWT using all points and leaf points only. Similar results have been obtained before for broadleaf species after applying an incidence angle correction to the intensity data at 1,550 nm (Zhu et al., 2015; Junttila et al., 2016). The correlations obtained in this study are relatively high for single wavelength data considering that no radiometric corrections have been applied to the data. Due to the shape and size of the spruce needles, an incidence angle correction would be impossible to apply because the laser footprint illuminates several needles at a time.

The spectral indices calculated from the 690, 905, and 1,550 nm wavelengths showed higher correlations with leaf EWT than the single wavelength data, which is in accordance with previous research (Junttila et al., 2016). The regression model of EWT and NDI_p70_690, 1,550_ showed a coefficient of determination of 0.82 for all points, while NDI_p70_905, 1,550_ was able to explain 89% of the changes in EWT. The better correlation of the combination of 1,550 and 905 nm with EWT can be explained by the sensitivity of the 905 nm wavelength to internal structure and dry matter content, a property needed in the estimation of EWT according to the literature (Ceccato et al., 2001). Additionally, the scanners providing measurements at 1,550 and 905 nm wavelengths were both developed by FARO and seem to employ similar algorithms for producing the discrete point clouds. It can be seen from the relationships between raw intensity and reflectance (Figure 1) that FARO scanners are more similar compared with the Leica, which could have affected our results.

Leaf points were used to develop linear regression models of the relationship between EWT and laser intensity features. The models showed that the most accurate prediction of EWT was achieved with the NDI_p80_905, 1,550_ feature with an *R*^2^ of 0.89. The accuracy of the prediction of EWT with segmented leaf points was the same as with all points but with a different feature. The best explanatory feature using all points was NDI_p70_690, 1,550_. This suggests that there are a lot of “noise” points still present in the point clouds considering the estimation of EWT in the lowest intensity percentiles because a higher percentile of the intensity distribution best explained EWT using leaf points. An intensity threshold could be applied to reduce the amount of noise points from the needles, but applying such a threshold greatly affects the distribution of the intensity values. Thus, a robust method would be needed to apply a threshold to different scans without losing valuable information regarding the intensity distribution.

The point density in each point cloud representing a seedling was reduced using random sampling to investigate the effect of point density on the estimation of EWT. The results showed that a lower scanning resolution could be used because the spectral indices calculated from the sparser point clouds still explained EWT almost as well as with maximum point density. After reducing the number of points down to 500 per seedling, the amount of variation explained was only slightly reduced. A more severe reduction in the amount of variance explained was present after the number of points was reduced to 250 per seedling. The NDI_p70_905, 1550_ index seemed to be more affected than NDI_p70_690, 1550_ by the reduction in point density.

A multiple regression model was developed with a laser intensity feature and length as predictors to estimate EWT. The inclusion of the length of the seedling in the regression model increased the accuracy of the estimation of EWT, although it should be pointed out that the length of the seedling varied only from 16.5 to 42 cm. Presumably, the inclusion of the length of the seedling is able to explain a part of the variation in EWT due to physiological alterations that occur during the life span of the needles. During the growing season, the specific leaf area (i.e., dry matter content per leaf area) increases due to accumulation of dry matter resulting in a lower EWT (Jach and Ceulemans, 2000).

The measurements in this study were conducted in a greenhouse, where environmental conditions were similar between measurements. Temperature and ambient light conditions did change a little between measurements depending on whether it was a sunny or cloudy day, but otherwise no alterations in range or background of the seedlings were present. The change in temperature and light conditions is a limitation that needs to be taken into account considering the applicability of the method for the forest environment. Here, we also found that stem points responded to changes in EWT, which is likely due to the thin bark of the seedlings. Mature trees have thicker bark; thus, the estimation of EWT with multispectral TLS likely requires segmentation or classification of laser returns to leaf and stem points.

The terrestrial laser scanners had slightly different technical parameters: The FARO scanners had similar specifications except for the laser beam diameter at output, which was 2.25 and 3 mm for the X330 and S120, respectively. The different specifications resulted in a different laser footprint at target but it seems that having the same laser footprint size at target is not crucial for calculating spectral indices because the correlations were high despite this factor. However, an airborne laser scanning measurement would result in much larger laser footprints, ~5–30 cm in diameter depending on the flying altitude, and a significantly lower point density. At these resolutions, the separation of woody and leaf material is practically impossible. The results obtained in this study show that the separation is not always necessary, but mature trees with large branches are likely to show a different response in terms of laser intensity.

The effect of incidence angle on spectral indices developed from multispectral laser scanning has been recently studied (Kaasalainen et al., 2016; Hancock et al., 2017). An NDI utilizing 1,063 and 1,545 nm wavelengths was concluded to not be sensitive to incidence angle effects when retrieving leaf moisture content. The combination of 905 and 1,550 nm wavelengths could be assumed to have similar properties due to the physical similarity of the wavelengths. Here, the needles had varying incidence angles and also varying receiving area because needles are not evenly spaced along the branches. Using an NDI calculated from the 905 and 1,550 nm wavelengths seems to reduce the effect of such confounding factors because using single wavelength data at 1,550 nm did not provide estimates of EWT as accurate as the spectral index did.

A commercially available multispectral laser scanner has been developed for airborne measurements, called Optech Titan (Teledyne Optech, Concord, Canada), which employs three wavelengths at 532, 1,064, and 1,550 nm. The combination of 1,064 and 1,550 nm wavelengths is interesting from the forest health mapping aspect, because these bands have been used before for EWT estimation (Gaulton et al., 2013). However, the different wavelengths of the Optech Titan have different footprints in terms of spatial distribution due to the instrument design (Morsy et al., 2016). The 1,064 nm channel is looking at nadir while the 1,550 nm channel is looking forward at 3.5° angle. This feature of the instrument presumably complicates the use of NDIs with the data, but the matter requires further investigation on the extent of the effect of varying spatial footprints at the canopy scale. A multispectral laser scanner for airborne measurements with overlapping footprints would be of great benefit in exploiting all the gains of the multispectral domain.

Based on the results, multisensor MS-TLS is capable of explaining most of the variation in leaf EWT of trees (*R*^2^ = 0.91) in a controlled environment. The combination of 905 and 1,550 nm wavelengths explained variation in EWT better than the combination of 690 and 1,550 nm, which is also supported by previous research (Ceccato et al., 2001). The high coefficients of determination in the estimation of EWT with spectral indices did not seem to require a high point density like we had in the original scans, but the scanning could be conducted at a lower resolution. Single trees measured from the sample plots provide a base for mapping and monitoring forests. Considering the small scale of our study and the larger scale of information that is required for monitoring declining forests and trees, we suggest that in the next step, tree-level EWT estimation in forest environment should be investigated using multispectral laser scanning at the 905 and 1,550 nm wavelengths and by classifying leaf and stem points.

## Author contributions

SJ designed the experiment, conducted data processing and statistical analysis, and wrote the manuscript. JS contributed to the laboratory analysis and writing of the manuscript. MV assisted in designing the experiment, statistical analysis, and the writing of the manuscript. RL contributed to the design of the experiment, data collection, and commented on the manuscript. HK contributed to the design of the experiment, gave technical advice, and commented on the manuscript. AK assisted with the measurement set-up and provided technical support. MH, HH, and JH arranged all the required resources, supervised the experiment, and participated in the writing of the manuscript. All of the authors have contributed to the finalizing of the manuscript.

### Conflict of interest statement

The authors declare that the research was conducted in the absence of any commercial or financial relationships that could be construed as a potential conflict of interest.
